# The influence of different forms of apple products on all-cause mortality in patients with hypertension

**DOI:** 10.3389/fnut.2024.1461196

**Published:** 2025-01-24

**Authors:** Chuang Sun, Yingying Chen, Yue Guan, Yiming Zeng, Jie Li, Liang Chen

**Affiliations:** ^1^Department of Cardiology, The Second Affiliated Hospital of Dalian Medical University, Dalian, China; ^2^Department of Internal Medicine, Ruijin-Hainan Hospital Shanghai Jiao Tong University School of Medicine (Hainan Boao Research Hospital), Qionghai, China

**Keywords:** apple consumption, apple products, hypertension, mortality, NHANES

## Abstract

**Objective:**

Apple consumption has a positive effect on human health. Some studies have shown that an appropriate amount of apple intake can reduce the incidence of hypertension. However, few studies have investigated whether eating different forms of apples has the same benefits as eating whole apples. This study is aimed to evaluate the effect of different forms of apple on all-cause mortality in patients with hypertension.

**Methods:**

The study included 2,368 patients with hypertension. All participants were followed up for at least 10 years. Cox regression model was constructed to analyze the correlation between apple, apple juice, and apple sauce consumption and all-cause mortality in patients with hypertension.

**Results:**

The consumption of apples 3–6 times/week was associated with a 48% reduction in the risk of all-cause mortality in patients with hypertension (HR = 0.52, 95% CI: 0.37–0.72, *p* < 0.001). However, the consumption of apple juice (HR = 1.02, 95% CI: 0.67–1.56, *p* = 0.930) and sauce (HR = 1.28, 95% CI: 0.59–2.74, *p* = 0.531) tended to increase the risk of death in patients with hypertension, although this study did not obtain a statistically result.

**Conclusion:**

Moderate consumption of whole apples is associated with a reduced risk of all-cause death in patients with hypertension, whereas apple juice and sauce may increase the risk of death.

## Introduction

As one of the most common diseases in the world, hypertension is an important risk factors for the global burden of the disease (1). Hypertension affects 1.5 billion people worldwide, which cause more than 10 million deaths each year, and is the leading cause of premature death worldwide (2, 3). Over time, this figure has increased each year. Therefore, early intervention to reduce global morbidity in patients with hypertension is particularly important for the development of worldwide health (4, 5). The first-line treatment recommended in most hypertension guidelines is lifestyle modification, with a healthy dietary pattern being particularly important and with an emphasis on fiber intake and fruit intake (5, 6).

Apples, one of the most widely produced and consumed fruit crops in temperate regions, are an important source of nutrients in human daily diets, providing many benefits to human health (7, 8). As the saying goes, an apple a day keeps the doctor away (9). Apples are rich in polyphenols, vitamins, cellulose and other bioactive phytochemicals. These ingredients can effectively reduce blood pressure, prevent lipid oxidation, reduce insulin resistance, regulate systemic inflammation, improve immune and vascular function, protect cells from oxidative damage, reduce platelet aggregation and antibacterial and antiviral activity, and inhibit cancer cell proliferation (9–12).

Previous studies have shown that eating apples may help reduce the risk of cardiovascular diseases, cancers, diabetes, obesity, and inflammation (9, 12, 13). With the advancement of industrial technology, for the convenience of consumption, transportation, and storage, an increasing number of apples are being processed into apple products. Therefore, it is necessary to understand whether processed apple can be used as substitutes for apples. This study aimed explore the relationship between apple, apple juice, and applesauce consumption and all-cause mortality in hypertensive patients.

## Materials and methods

### Data source and study population

The dataset utilized in this research originated from the National Health and Nutrition Examination Survey (NHANES). The ethical integrity of this study was upheld through the approval granted by the Ethics Review Committee of the National Center for Health Statistics, ensuring adherence to ensure that human subjects are treated with the utmost ethical consideration. Consent was obtained in writing from all participants. Any researcher can download the data from the website for free.^1^ All experiments involved in this paper are performed in accordance with relevant guidelines and regulations.

**Figure 1:**
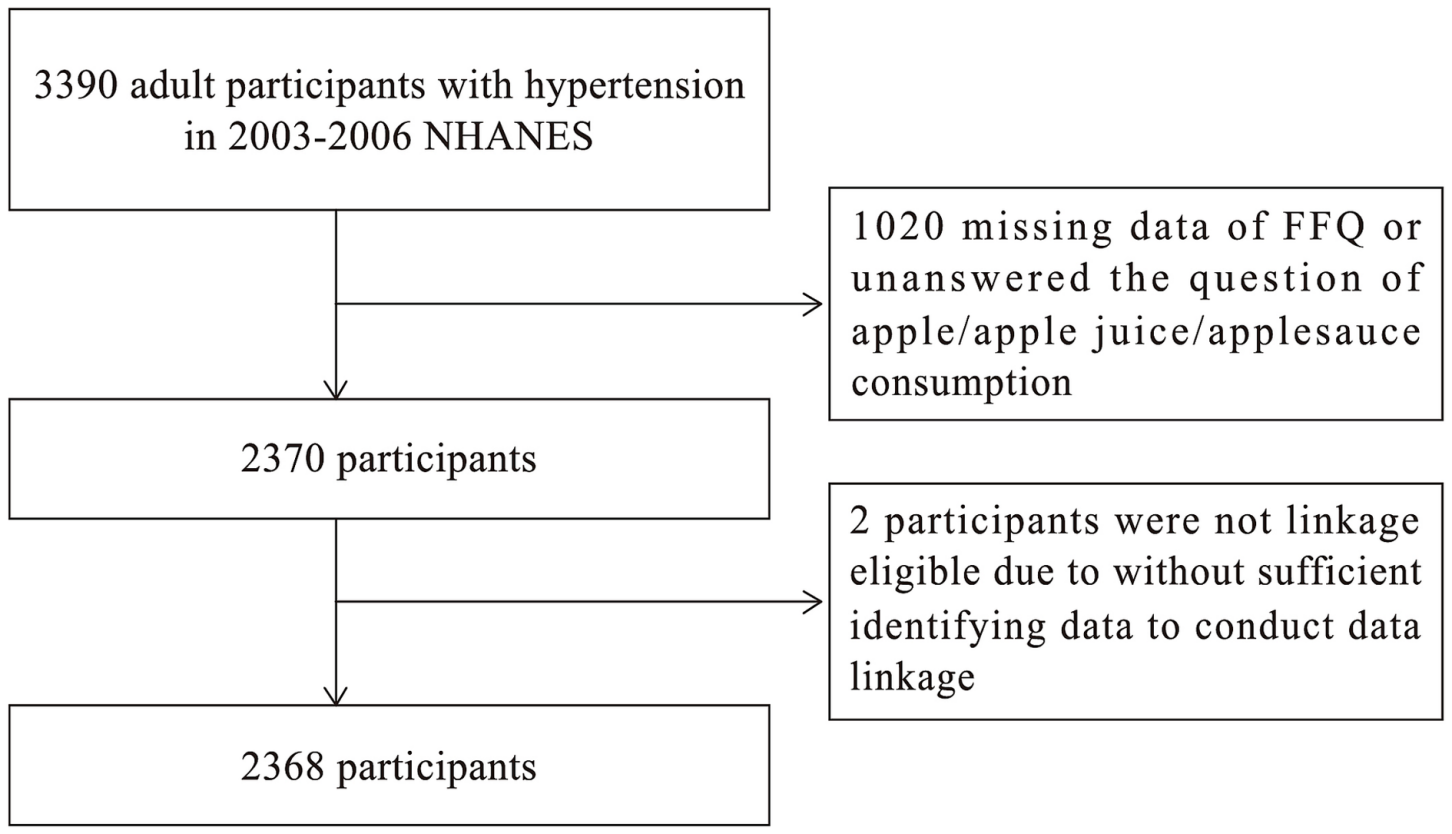
Flowchart of participant of selection.

Between the years 2003 and 2006, the study identified 3,390 hypertensive patients using a computer-aided interviewing system and questionnaires. All participants lived in the United States at the time of the interview. Individuals who did not complete the Frequency of Consumption Questionnaire (FFQ) were excluded from the study (*n* = 1,020), as were those who did not have sufficient information related to the National Death Index (NDI) (*n* = 2). The final number of subjects included in this study was 2,368, as illustrated in Figure 1.

### Assessment of consumption of apples, apple juice and apple sauce

The food frequency questionnaire (FFQ) is an instrument that collects frequency information based on the past 12 months’ food intake, including information on consumer habits related to fruit. Participants were asked, “How often do you eat apples/apple juice/apple sauce?” to inquire about their average apple and apple product intake over the past year. Fruit consumption data were obtained by FFQ, and the intake levels were categorized as follows: not eating, <1 times/week, 1–2 times/week, 3–6 times/week, or ≧ 1 times/day.

### Survival follow-up

The NDI data were released by the National Center for Health Statistics, a US government agency, which recorded the death information of NHANES participants. It can be found at http://www.cdc.gov/nchs/nhanes/index.htm. All participants in this study were followed up for at least 10 years.

### General information collection

The baseline characteristics of the patients were gathered utilizing a computerized personal interview platform and a structured questionnaire, encompassing demographic attributes as well as health status indicators. These encompassed smoking habits, alcohol intake, and the existence of hyperlipidemia, diabetes mellitus, cardiac conditions, and cerebrovascular accidents. The demographic profile comprehended gender, age, race, educational attainment, and the ratio of family income to the poverty. Individuals classified as smokers were those who had a lifetime history of consuming at least 100 cigarettes, indicating a degree of tobacco use that transcended casual experimentation. Similarly, an alcohol drinker was defined as someone who consumed at least 12 units of alcohol during the previous 12-month period, marking a level of consumption sufficient to be considered regular or moderate. The identification of hypercholesterolemia, diabetes, cardiac disorders, and stroke relied on the participants’ self-reported information. Specifically, the diagnosis of cardiac disease was confirmed when individuals responded positively to any of the following inquiries: “Have you previously received a diagnosis of congestive heart failure /coronary heart disease /angina pectoris /myocardial infarction?”

### Statistical analysis

The nonlinear associations between the consumption of apples, apple juice, and apple sauce and the risk of all-cause death in hypertensive patients were analyzed by smoothing curves based on a restricted cubic spline function. All covariates were evaluated for potential collinearity and found to have variance inflation factors below 5. Subsequently, Cox proportional hazard models were constructed to investigate the impact of apple, apple juice, and apple sauce consumption on mortality. In order to ascertain the consistency of the results in the presence of different diseases in combination, stratified analyses were employed.In addition, KM survival curves further elucidated the relationship between apple intake and mortality. To adjust the confounders stepwise, three models were developed. No variables were adjusted in Model 1. Model 2 was adjusted for demographic data (including gender, age, and race). In Model 3, in addition to demographic information, the covariates that changed estimates of the effect of apple on hypertension by more than 10% were also adjusted (including gender, age, race, education level, ratio of family income to the poverty, smoking, hypercholesterolemia, diabetes, heart disease and stroke). An analysis of the data was conducted using Empower software, and we considered *p* values less than 0.05 to be statistically significant.

## Results

### Characteristics of the study population

The study finally included 2,368 hypertensive patients (Figure 1). In Table 1, participants are described by their baseline characteristics. After 10 years of follow-up, 1750 patients were alive, and 618 died. The surviving cohort demonstrated distinct characteristics when compared to the non-surviving group, notably featuring a younger age distribution, a higher proportion of females, and potentially a superior health profile, marked by an absence of hyperlipidemia, diabetes, cardiac ailments, and cerebrovascular events. Furthermore, statistically meaningful disparities emerged in terms of racial composition, educational level, the ratio of family income to poverty threshold, and smoking history between the two groups.

**Table 1:** Baseline characteristics of participants.

	All	Survival	All-cause death	*p*
Number	2,368	1750	618	
Age	63.0 (50.0–73.0)	60.0 (46.0–69.0)	75.0 (65.2–80.0)	<0.001
Gender				<0.001
Male	1,123 (47.4)	782 (44.7)	341 (55.2)	
Female	1,245 (52.6)	968 (55.3)	277 (44.8)	
Race				<0.001
Mexican American	344 (14.5)	272 (15.5)	72 (11.7)	
Other Hispanic	39 (1.6)	33 (1.9)	6 (1.0)	
Non-Hispanic White	1,361 (57.5)	950 (54.3)	411 (66.5)	
Non-Hispanic Black	547 (23.1)	430 (24.6)	117 (18.9)	
Other race	77 (3.3)	65 (3.7)	12 (1.9)	
Education level				<0.001
﹤High school	694 (29.3)	462 (26.4)	232 (37.5)	
High school graduate or general equivalency diploma	655 (27.7)	482 (27.5)	173 (28.0)	
﹥High school	1,019 (43.0)	806 (46.1)	213 (34.5)	
Ratio of family income to poverty				<0.001
≤1	346 (14.6)	252 (14.4)	94 (15.2)	
1–3	1,037 (43.8)	709 (40.5)	328 (53.1)	
﹥3	876 (37.0)	717 (41.0)	159 (25.7)	
Unknown	109 (4.6)	72 (4.1)	37 (6.0)	
Alcohol use				0.393
No	901 (38.0)	657 (37.5)	244 (39.5)	
Yes	1,467 (62.0)	1,093 (62.5)	374 (60.5)	
Smoking				<0.001
No	1,125 (47.5)	894 (51.1)	231 (37.4)	
Yes	1,243 (52.5)	856 (48.9)	387 (62.6)	
Hypercholesterolemia				0.013
No	1,217 (51.4)	926 (52.9)	291 (47.1)	
Yes	1,151 (48.6)	824 (47.1)	327 (52.9)	
Diabetes				<0.001
No	1887 (79.7)	1,443 (82.5)	444 (71.8)	
Yes	481 (20.3)	307 (17.5)	174 (28.2)	
Heart disease				<0.001
No	1886 (79.6)	1,501 (85.8)	385 (62.3)	
Yes	482 (20.4)	249 (14.2)	233 (37.7)	
Stroke				<0.001
No	2,169 (91.6)	1,660 (94.9)	509 (82.4)	
Yes	199 (8.4)	90 (5.1)	109 (17.6)	
Apple				<0.001
Never	237 (10.0)	149 (8.5)	88 (14.2)	
﹤1 times/week	1,293 (54.6)	970 (55.4)	323 (52.3)	
1–2 times/week	409 (17.3)	308 (17.6)	101 (16.3)	
3–6 times/week	333 (14.1)	261 (14.9)	72 (11.7)	
≧1 times/day	96 (4.1)	62 (3.5)	34 (5.5)	
Apple Juice				<0.001
Never	850 (35.9)	578 (33.0)	272 (44.0)	
﹤1 times/week	1,141 (48.2)	882 (50.4)	259 (41.9)	
1–2 times/week	170 (7.2)	133 (7.6)	37 (6.0)	
3–6 times/week	121 (5.1)	96 (5.5)	25 (4.0)	
≧1 times/day	86 (3.6)	61 (3.5)	25 (4.0)	
Applesauce				<0.001
Never	824 (34.8)	643 (36.7)	181 (29.3)	
﹤1 times/week	1,279 (54.0)	941 (53.8)	338 (54.7)	
1–2 times/week	171 (7.2)	107 (6.1)	64 (10.4)	
3–6 times/week	75 (3.2)	48 (2.7)	27 (4.4)	
≧1 times/day	19 (0.8)	11 (0.6)	8 (1.3)	

A median (Q1, Q3) is used for continuous variables, and a percentage (%) is used for categorical variables.

### Association of apple products consumption with all-cause mortality

**Figure 2:**
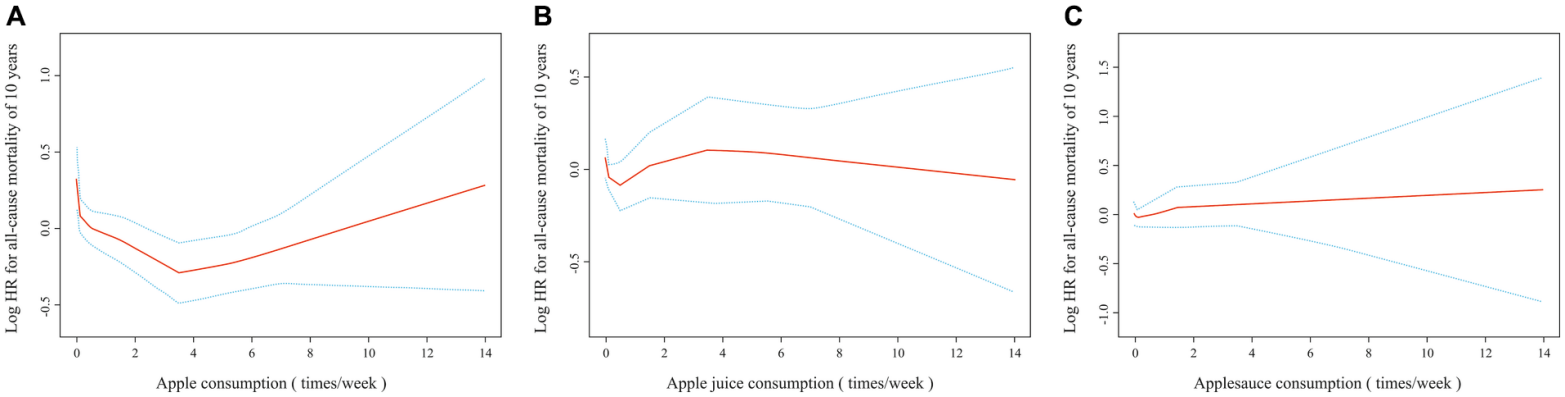
Spline smoothing plots between apple products and all-cause mortality. **(A)** Apple consumption and all-cause mortality. **(B)** Apple juice. **(C)** Applesauce. A full adjustment has been made to the HR for gender, age, race, education level, ratio of family income to the poverty, smoking, hypercholesterolemia, diabetes, heart disease and stroke.

Smoothing curves based on the restrictive cubic spline function were drawn (Figure 2). The results showed that there was a nonlinear association between apple intake and all-cause mortality. With increasing apple intake, the mortality rate decreased, but above a threshold, the mortality rate did not decline further as the intake continued to increase. However, regardless of changes in the intake of apple juice and applesauce, all-cause mortality did not change significantly. As shown in Table 2, after fully adjusting for confounding factors, compared with not eating apples, the consumption of apples on three to six occasions per week was found to be significantly associated with a reduced risk of mortality (HR = 0.52, 95% CI: 0.37–0.72, *p* < 0.001). However, this was not observed for other intakes of apples.

**Table 2:** Cox regression analysis.

	Number	Model 1	Model 2	Model 3
		HR (95%CI) *p*	HR (95%CI) *p*	HR (95%CI) *p*
Apple				
Never	237	Ref.	Ref.	Ref.
﹤1 times/week	1,293	0.61 (0.48, 0.77) <0.001	0.70 (0.55, 0.89) 0.003	0.79 (0.62, 1.02) 0.068
1–2 times/week	409	0.60 (0.45, 0.80) 0.001	0.63 (0.47, 0.84) 0.002	0.77 (0.57, 1.04) 0.088
3–6 times/week	333	0.52 (0.38, 0.71) <0.001	0.48 (0.35, 0.65) <0.001	0.52 (0.37, 0.72) <0.001
≧1 times/day	96	0.91 (0.61, 1.35) 0.649	0.71 (0.47, 1.05) 0.089	0.83 (0.55, 1.26) 0.383
Apple juice				
Never	850	Ref.	Ref.	Ref.
﹤1 times/week	1,141	0.66 (0.56, 0.79) <0.001	0.78 (0.66, 0.93) 0.005	0.84 (0.71, 1.01) 0.062
1–2 times/week	170	0.64 (0.45, 0.90) 0.010	0.73 (0.52, 1.04) 0.081	0.87 (0.60, 1.24) 0.431
3–6 times/week	121	0.62 (0.41, 0.93) 0.020	0.91 (0.60, 1.37) 0.650	0.91 (0.59, 1.40) 0.662
≧1 times/day	86	0.89 (0.59, 1.34) 0.581	1.00 (0.66, 1.52) 0.988	1.02 (0.67, 1.56) 0.930
Applesauce				
Never	824	Ref.	Ref.	Ref.
﹤1 times/week	1,279	1.23 (1.02, 1.47) 0.026	0.88 (0.73, 1.06) 0.172	0.97 (0.80, 1.18) 0.784
1–2 times/week	171	1.85 (1.39, 2.46) <0.001	1.01 (0.75, 1.36) 0.930	1.07 (0.79, 1.46) 0.648
3–6 times/week	75	1.85 (1.24, 2.78) 0.003	1.01 (0.67, 1.52) 0.970	1.19 (0.78, 1.83) 0.419
≧1 times/day	19	2.18 (1.07, 4.43) 0.031	1.28 (0.63, 2.61) 0.495	1.28 (0.59, 2.74) 0.531

Model 1: Not adjusted.

Model 2: Adjusted for gender, age, race.

Model 3: Adjusted for gender, age, race, education level, ratio of family income to the poverty, smoking, hypercholesterolemia, diabetes, heart disease and stroke.

It is worth noting that, without any adjustment, moderate intake of apple juice can reduce the risk of death compared with the reference group. Apple sauce consumption was positively associated with death risk. However, after fully adjusting for confounding factors, the analysis revealed no statistically significant correlation between apple juice and applesauce intake and mortality.

### Association of apples consumption with all-cause mortality

**Figure 3:**
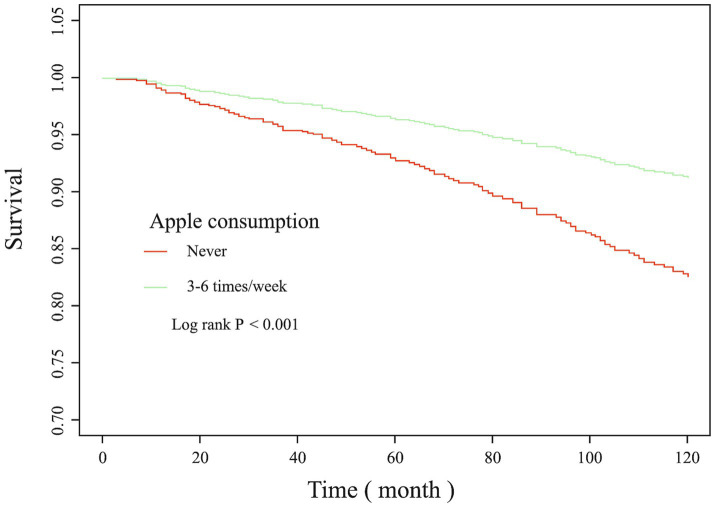
KM survival curve. Adjusted for gender, age, race, education level, ratio of family income to the poverty, smoking, hypercholesterolemia, diabetes, heart disease and stroke.

Regression analysis revealed that appropriate apple intake could reduce the risk of death. This study further investigated the association between apple consumption and mortality. The results of subgroup analysis (Table 3) showed that for the hypertensive population, the correlation between apple intake and all-cause mortality risk was not affected by the presence of other diseases (P interaction were all greater than 0.05). As shown in Figure 3, after fully adjusting for confounding factors, compared with people who never eat apples, people who ate apples 3–6 times per week were associated with a higher survival rate (Log rank *p* < 0.001). Further analysis of the causes of death, the result showed that 3–6 times/week apple intake could reduce the mortality by various diseases (*p* = 0.007), the results are shown in Supplementary Table 1.

**Table 3:** Stratified analysis.

	Number	HR (95%CI)					P inter
		Never	﹤1 times/week	1–2 times/week	3–6 times/week	≧1 times/day	
Hypercholesterolemia							0.868
No	1,217	Ref.	0.79 (0.56, 1.10)	0.85 (0.56, 1.29)	0.57 (0.36, 0.89)	0.87 (0.46, 1.68)	
Yes	1,151	Ref.	0.77 (0.53, 1.13)	0.68 (0.43, 1.06)	0.45 (0.27, 0.73)	0.77 (0.44, 1.35)	
Diabetes							0.576
No	1887	Ref.	0.86 (0.64, 1.15)	0.82 (0.58, 1.17)	0.55 (0.37, 0.81)	1.05 (0.63, 1.74)	
Yes	481	Ref.	0.57 (0.34, 0.94)	0.61 (0.33, 1.12)	0.37 (0.20, 0.69)	0.53 (0.25, 1.10)	
Heart disease							0.923
No	1886	Ref.	0.78 (0.56, 1.08)	0.73 (0.49, 1.08)	0.51 (0.33, 0.78)	0.92 (0.54, 1.54)	
Yes	482	Ref.	0.82 (0.55, 1.22)	0.90 (0.56, 1.46)	0.52 (0.30, 0.89)	0.81 (0.39, 1.65)	
Stroke							0.154
No	2,169	Ref.	0.76 (0.57, 1.01)	0.69 (0.49, 0.97)	0.52 (0.36, 0.74)	0.98 (0.62, 1.54)	
Yes	199	Ref.	0.96 (0.55, 1.68)	1.27 (0.64, 2.55)	0.37 (0.14, 0.97)	0.51 (0.16, 1.61)	

Adjusted for gender, age, race, education level, ratio of family income to the poverty, smoking, hypercholesterolemia, diabetes, heart disease and stroke (in addition to the stratification factor itself).

## Discussion

This study showed that in patients with hypertension, the consumption of apples on three to six occasions per week was found to be significantly associated with a reduced risk of mortality, whereas the consumption of processed apple products (apple juice and applesauce) does not have a similar effect. This is the first observational study to simultaneously investigate the relationship between intake of whole apples and processed apple products and all-cause mortality in hypertensive patients.

Previous studies have revealed that the prognosis of hypertension in conjunction with other diseases, including hyperlipidemia, diabetes mellitus, heart disease, and stroke, differs (14–17). Previous studies have shown that moderate apple consumption can reduce the risk of hypertension, diabetes, cardiovascular disease, stroke (18). Bogri et al. reported that eating apples four or more times a week reduced the risk of hypertension (19). Another study by Muraki et al. pointed out that eating three servings of apples per week can reduce the risk of type 2 diabetes (20). Daily intake of 100–150 grams of apple reduces the risk of cardiovascular disease (21). Susanna et al. reported that apple consumption could reduce the risk of stroke by 11% (22). Nevertheless, the subgroup analysis of this study revealed that apple consumption was associated with improved prognosis in hypertensive patients, irrespective of the presence of other comorbidities, including hyperlipidemia, diabetes, heart disease, and stroke. It can be reasonably deduced that for all populations afflicted with hypertension, the incorporation of apples into their daily diet may prove to be of considerable prognostic benefit.

To date, few studies have investigated the association between apple consumption and all-cause mortality. The results of the cohort study by Jonathan et al. indicated that among older women, higher apple intake was associated with a lower risk of death after multivariate adjustment (HR = 0.89, 95% CI: 0.81, 0.97) (23). The present study yielded analogous results, demonstrating that appropriate whole apples intake is negatively associated with mortality.

The health benefits of fruit have been recognized,nevertheless, the evidence pertaining to the health effects of fruit juices is both limited and inconsistent. In a meta-analysis study, Pan et al. found that the relationship between 100% fruit juice consumption and all-cause mortality was inconclusive (24). In a prospective cohort study involving 198,285 participants, Anderson et al. found a significant inverse dose–response relationship between fruit or vegetable juice consumption and all-cause mortality, however, this association did not persist after additional adjustment for diet quality score (25). This finding is similar to that of the present study. Compared with no consumption, there was a negative association between apple juice consumption and mortality when apple juice was consumed at other frequencies, but after fully adjusting for confounding factors, there was no significant association between apple juice consumption and all-cause mortality. However, Zhang found that compared to non-juice drinkers, the intake of 250 grams or more of 100% fruit juice per day has been demonstrated to elevate the risk of mortality by 30% (HR = 1.30, 95%CI: 1.11–1.52), and the analysis of total fruit juice also showed similar results (HR = 1.28, 95%CI: 1.09–1.49) (26). Xu et al. also found that the consumption of fruit juice was found to be positively correlated with the risk of all-cause mortality (HR = 1.26, 95% CI: 1.12–1.41, *p* < 0.001), while no significant association was found in male participants (27). The differences in the study results may be mainly attributed to differences in the study populations, the different quantification methods of juice consumption, the adjustment of confounding variables, the follow-up time, and cohort characteristics. Therefore, more large-sample cohort studies with long-term follow-up are still needed to verify the relationship between juice intake and all-cause mortality, especially the association between apple juice and all-cause mortality.

To our knowledge, no studies have assessed the association between applesauce intake and mortality risk. The results of this study fill this gap. Our study found that compared to patients with hypertension who never consumed applesauce, those who consumed applesauce had an increased risk of death, but after fully adjusting for confounding factors, although a similar trend was observed, it was not statistically significant.

Apples were the main source of polyphenols in the diet (8). Compared with other fruits, apples may contain more free phenols, which are more easily absorbed and utilized by the human body (28, 29). As strong antioxidants, apple polyphenols can effectively prevent the oxidation of lipids and DNA, scavenge free radicals, inhibit the generation of related reactive oxygen species, inhibit tumor cell proliferation, regulate gene expression, induce cell apoptosis, change the conduction of signaling pathways, and strengthen the immune system (11, 12, 30, 31). The polyphenol contents are different in different anatomical parts of apples (32). Studies have shown that the phenolic content of in apple peel is two to six times greater than that in apple pulp (29). Apple polyphenols are mainly divided into phenolic acids and flavonoids (33). Phenolic acids mainly include gallic acid, protocatechuic acid, vanillic acid, syringic acid, quinic acid, caffeic acid, chlorogenic acid, p-coumaroyl quinic acid and pcoumaric acid (33). There are four main subclasses of flavonoids: flavonols, flavanols-3-ols, anthocyanins and dihydrochalcones (33). There are greater levels of phenolic acids in apple peels than in apple flesh (34, 35). Flavonols account for approximately 71 to 90% of the total flavonoid content of apples. Furthermore, the highest content of flavonols is quercetin glycoside (containing quercetin 3-glycoside), which has extremely high in antioxidant properties and is mainly found in the fruit skin (33). Flavan-3-ols are mainly found in the apple peel and pulp. Anthocyanins are the most abundant in apple peels. Dihydrochalcone levels are higher in apple seeds and pomace (10). Owing to differences in polyphenol content, the antioxidant activity of apple peels is two to six times higher than that of apple pulp (29). During the processing of processed apple products, such as applesauce and apple juice, the apple peel is lost, which significantly reduces the phenolic content and thus the antioxidant activity (12, 33). Apple juice and applesauce may require crushing of apples during processing, which leads to the oxidation of apples and further reduction of phenolic substances. In addition, the processing generates waste called apple pomace (10). Lianosides, chlorogenic acid, epicatechin and quercetin glycosides can be isolated from apple pomace. These substances have high antioxidant activity, indicating that apple pomace may have certain nutritional value and commercial use (29). The processing of apples results in a notable decline in phenolic content. This could provide an explanation for the observed phenomenon that apple consumption can lead to significantly lower mortality, but processed apple products may not.

Apples are rich in a variety of vitamins, especially vitamin C, which is also a strong antioxidant (10). Vitamin C not only scavenges free radicals, but also increases the release of nitric oxide, thereby improving vascular endothelial function. Vitamin C also promotes the regeneration of vitamin E, protects cells from free radical damage, and delays aging (36). However, vitamin C is relatively unstable and is easily damaged by water, alkali, heat, light and oxygen. Therefore, the vitamin C content of whole apples is usually greater than that of processed apples (36).

The dietary fiber in apples is also associated with a reduced risk of various diseases, including cardiovascular diseases, cancer, diabetes, obesity, gastrointestinal diseases, etc. (37). Apple peels contain more cell walls and fibers than apple pulp, and have a significantly greater dietary fiber content than pulp (38). Therefore, apple processing affects the integrity of the apple cell wall and fiber content and changes the bioavailability of phytochemicals (38). Therefore, this study recommended the consumption of whole apples.

Moreover, many additives are used in the processing of apples to prevent fruit deterioration and extend the shelf life of foods, which may affect human health [35–37]. Edible additives may increase the risk of cardiovascular diseases, diabetes and cancer in patients (39–41). In addition, apple juice and applesauce contain less fiber, which results in a higher glycemic index (42). Moreover, too much sugar is often added to apple products to improve the taste. The free sugars in fruit juice are approximately the same as those in sugar-sweetened drinks (43). Excessive sugar intake can increase the risk of diabetes and induce cardiovascular and cerebrovascular diseases, which are major causes of death in patients with hypertension (44). Apple processed products also use food packaging, of which perfluoroalkyl and polyfluoroalkyl substances are the major components (45). Studies have shown that perfluoroalkyl and polyfluoroalkyl substances increase the risk of hypertension and pose challenges to blood pressure control in hypertensive patients (45). Therefore, chemical substances during apple processing can weaken the benefits of apples for the human body, thus increasing the risk of death in patients with hypertension.

Apple is an easy-to-obtain, inexpensive fruit that is widely distributed, easy to store, and has high nutritional value. Using the NHANES data, this study revealed that apples could significantly reduce the risk of all-cause death in patients with hypertension, which provides evidence supporting the dietary choices of patients with hypertension. In addition, the consumption of processed apple products is not recommended because they may pose potential risks to humans, especially those with hypertension. The consumption of applesauce has been found to increase the risk of death in hypertensive patients, although the results of this study did not yield statistically significant positive findings in this regard.

### Weaknesses

This study has some limitations. The participants in this study were derived from NHANES, which, while nationally representative, may not generalize to other countries. The diagnosis of hypertension in this study was based on self-reports by participants, and many patients may not have been diagnosed because of the absence of obvious clinical symptoms, potentially leading to underreporting of hypertension. Although we have made efforts to consider all potential confounding variables and mitigate the impact of covariates, factors such as individual metabolic status and the possibility that many participants may consume both fruits and fruit products simultaneously cannot be completely excluded.

## Conclusion

Moderate consumption of apples can significantly reduce the mortality risk in hypertensive patients, whereas apple juice and applesauce may pose potential mortality risks. This study recommends the consumption of whole fresh apples for patients with hypertension.

## Data Availability

Publicly available datasets were analyzed in this study. This data can be found at: National Health and Nutrition Examination Survey (https://www.cdc.gov/nchs/nhanes/index.htm).

## Footnotes

^1^ https://www.cdc.gov/nchs/nhanes/index.htm
