# Supplementary material for: The influence of different forms of apple products on all-cause mortality in patients with hypertension
Source: Front Nutr. 2025 Jan 24;11:1461196. doi: 10.3389/fnut.2024.1461196 (PMC11802416; doi:10.3389/fnut.2024.1461196)

**SUPPLEMENTARY TABLE 1. Cause of death.**

|  | **All** | **Apple Consumption** | | | **P** |
| --- | --- | --- | --- | --- | --- |
| **Number** | **2368** | **Never** | **3-6 times/week** | **Other frequencies** |  |
| **Survival** | 1750 (73.9) | 149 (62.9) | 261 (78.4) | 1340 (74.5) | 0.007 |
| **Death of heart diseases** | 187 (7.9) | 25 (10.5) | 18 (5.4) | 144 (8.0) |  |
| **Death of malignant neoplasms** | 125 (5.3) | 14 (5.9) | 16 (4.8) | 95 (5.3) |  |
| **Death of chronic lower respiratory diseases** | 44 (1.9) | 11 (4.6) | 5 (1.5) | 28 (1.6) |  |
| **Death of accidents** | 12 (0.5) | 1 (0.4) | 0 (0.0) | 11 (0.6) |  |
| **Death of cerebrovascular diseases** | 39 (1.6) | 7 (3.0) | 5 (1.5) | 27 (1.5) |  |
| **Death of alzheimer’s disease** | 18 (0.8) | 1 (0.4) | 3 (0.9) | 14 (0.8) |  |
| **Death of diabetes** | 27 (1.1) | 2 (0.8) | 3 (0.9) | 22 (1.2) |  |
| **Death of influenza and pneumonia** | 15 (0.6) | 4 (1.7) | 2 (0.6) | 9 (0.5) |  |
| **Death of nephritis, nephrotic syndrome and nephrosis** | 21 (0.9) | 5 (2.1) | 4 (1.2) | 12 (0.7) |  |
| **Death of all other causes** | 130 (5.5) | 18 (7.6) | 16 (4.8) | 96 (5.3) |  |

**SUPPLEMENTARY FIGURE 1. ROC curve**


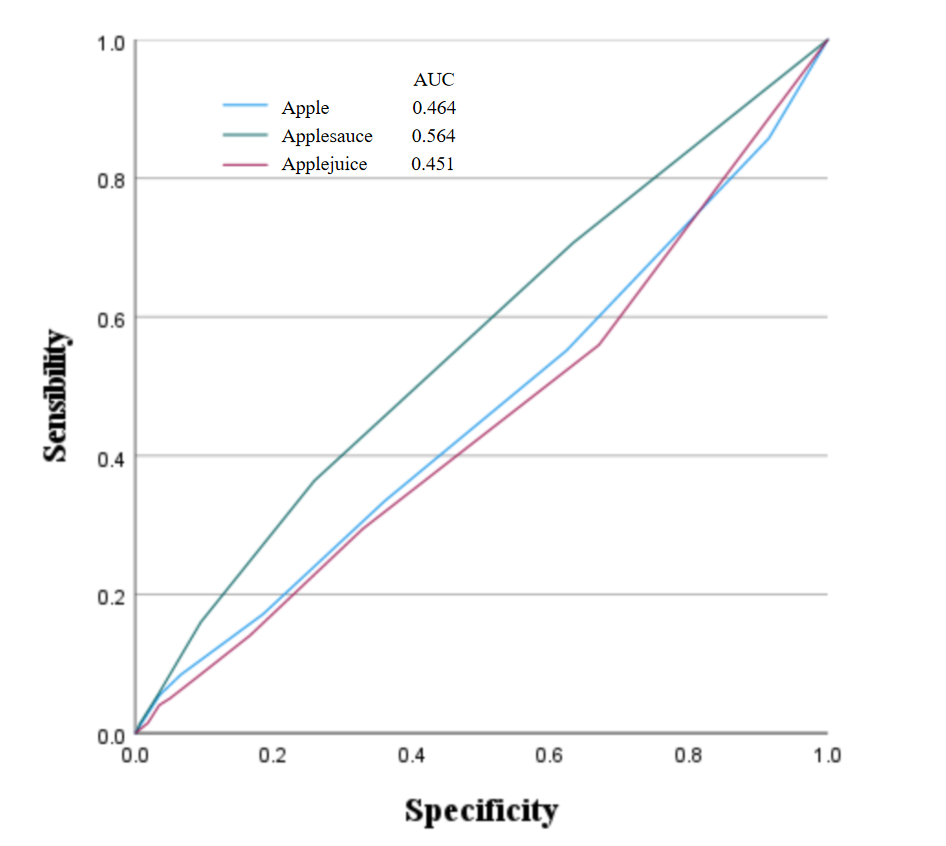

Supplement: Supplementary file 1 [file Table_1.DOCX]
